# Cellulose intrafibrillar mineralization of biological silica in a rice plant

**DOI:** 10.1038/s41598-021-87144-8

**Published:** 2021-04-12

**Authors:** Eri Nakamura, Noriaki Ozaki, Yuya Oaki, Hiroaki Imai

**Affiliations:** 1 Department of Applied Chemistry, Faculty of Science and Technology, Keio University, 3-14-1 Hiyoshi, Kohoku-ku, Yokohama, 223-8522 Japan; 2 Department of Biotechnology, Faculty of Bioresource Sciences, Akita Prefectural University, 241-438 Kaidobata-Nishi, Nakano Shimoshinjo, Akita, 010-0195 Japan

**Keywords:** Biomineralization, Plant development, Nanoparticles, Nanostructures, Scanning electron microscopy, Polysaccharides

## Abstract

The essence of morphological design has been a fascinating scientific problem with regard to understanding biological mineralization. Particularly shaped amorphous silicas (plant opals) play an important role in the vital activity in rice plants. Although various organic matters are associated with silica accumulation, their detailed functions in the shape-controlled mineralization process have not been sufficiently clarified. In the present study, cellulose nanofibers (CNFs) were found to be essential as a scaffold for silica accumulation in rice husks and leaf blades. Prior to silicification, CNFs ~ 10 nm wide are sparsely stacked in a space between the epidermal cell wall and the cuticle layer. Silica nanoparticles 20–50 nm in diameter are then deposited in the framework of the CNFs. The shape-controlled plant opals are formed through the intrafibrillar mineralization of silica nanoparticles on the CNF scaffold.

## Introduction

In nature, organisms produce various inorganic materials with precisely controlled morphologies from a limited selection of ubiquitous elements, such as calcium, silicon, carbon, and oxygen, under ambient conditions^1^. The morphological design is a critically important aspect of biological mineralization processes with regard to the emergence of specific functions. Biogenic amorphous silica is a typical biomineral that is widely observed in diatoms^2–7^, marine sponges^8–10^, and some higher plants^11–15^. Silica frustules of diatoms and skeletons of marine sponges have adequately designed hierarchical architectures^4,6,9^ and provide excellent mechanical and optical properties^16,17^. Rice plants accumulate large amounts of amorphous silicas called plant opals on the surface and inside of leaf blades, stems, and husks^14,15^. Recently, millimeter-scale plant opals were reported to be composed of silica nanoparticles (Figure S1 in the Supporting Information (SI)) and to have specific mechanical and optical properties depending on their structure and morphology^18–20^.

The formation of biosilicas is commonly ascribed to the polycondensation of orthosilicic acid in organisms under an ambient pressure and room temperature. The in vivo silicification process of rice plants through transpiration is generally controlled by organic matter, including proteins in cell walls^21^. According to previous studies on diatoms^22^, the diatom silica frustules contain a unique peptide, called silaffin, that is bound to long-chain polyamines. A particular repeating structure of the amino acid sequence of silaffin is involved in silica polymerization^23^. An assembly of the peptides increases the density of positively charged amino groups and cationic long-chain polyamines, and thus attracts negatively charged silicic acid^7^. Silica skeletons of marine sponges have been found to contain a protein called glassine^24^. This protein has a silicic acid polymerization activity with a charge relay effect that is associated with the alternating sequence of acidic amino acids and basic amino acids^25^. The promoted polymerization was confirmed by silicification with an artificial peptide modified with long-chain polyamines as a molecular template^26,27^. A protein that promotes silica polymerization in the apoplast was discovered in the cytoplasm of a higher plant, sorghum^28^. The amino acid sequence of the protein in sorghum contains a lysine-rich domain similar to silaffin and a histidine- and aspartic acid–rich domain similar to glassine. These proteins and polyamines contained in diatoms, sponges, and higher plants are regarded as a molecular-level template for the promotion of silica polymerization.

The macroscopic morphology of biosilicas is precisely designed in the biological bodies. A scaffold or a spatial template is commonly required beside the molecular templates to construct the controlled macroscopic shapes of biosilicas. Silicification of diatoms occurs in a membrane-bound compartment, the silica deposition vesicle (SDV), as a spatial template^29^. A particular morphology is formed through the polymerization of silicic acid in the SDV. The silica skeleton of sponges is composed of biosilicas formed around a fiber of silicatein playing a dual role as a molecular template and a scaffold^30^. In general, the presence of organic scaffolds is important for the production of macroscopically shape-controlled bodies of biogenic inorganic–organic composite materials.

Vertebrate bones and teeth are comprised of hydroxyapatite nanocrystals that are grown on the scaffold of collagen fibers. Chitin fibers are utilized as a scaffold for the deposition of amorphous calcium carbonate (ACC) in the gastrolith of crayfish^31^. Soluble proteins contained in the gastrolith were suggested to be involved in the ACC formation with the fibrous scaffold^32^. However, our understanding of the shape-controlled silicification mechanism with a spatial template for the production of particular shapes has been insufficient with regard to biosilicas. Polysaccharides, which are the main components of cell walls, are a candidate for a scaffold for silicification in higher plants. Callose, which is composed of glucose residues, was found to accumulate silica in rice and horsetail plants^33,34^. A mixed-linkage glucan consisting of glucosyl residues was reported to be utilized for the accumulation of silica^35^. Despite the efforts in previous studies, unfortunately, the essential role of polysaccharides as a scaffold for the silicification process remains unknown.

In the present article, we focus on an organic fibrous structure that is involved in silica particles in a rice plant. Our previous research revealed that rice plant opals are comprised of 20–50 nm silica particles^18^. Their mechanical properties were found to be controlled by their packing density. Here, we study the essence of cellulose nanofibers (CNFs) as a scaffold for the accumulation of silica nanoparticles through detailed observation of immature and mature rice husks and leaf blades. Although a fibrous substance was observed in silica bodies of orchard grass^36^ and horsetail plants^37^, the components and interaction with biosilicas were not discussed on the basis of experimental evidence. We clarified the formation of CNFs prior to silicification in the previous stage of the silicification. The accumulation of silica nanoparticles is deduced to be controlled by the CNF scaffold. The template function of the scaffold would be a key to the morphological design of plant opals. This understanding of the biogenic intrafibrillar silicification mechanism is extremely important in terms of gramineous crop cultivation and the biomimetic engineering of nanostructured materials.

## Results and discussion

### Plant opals in rice husks

**Figure 1:**
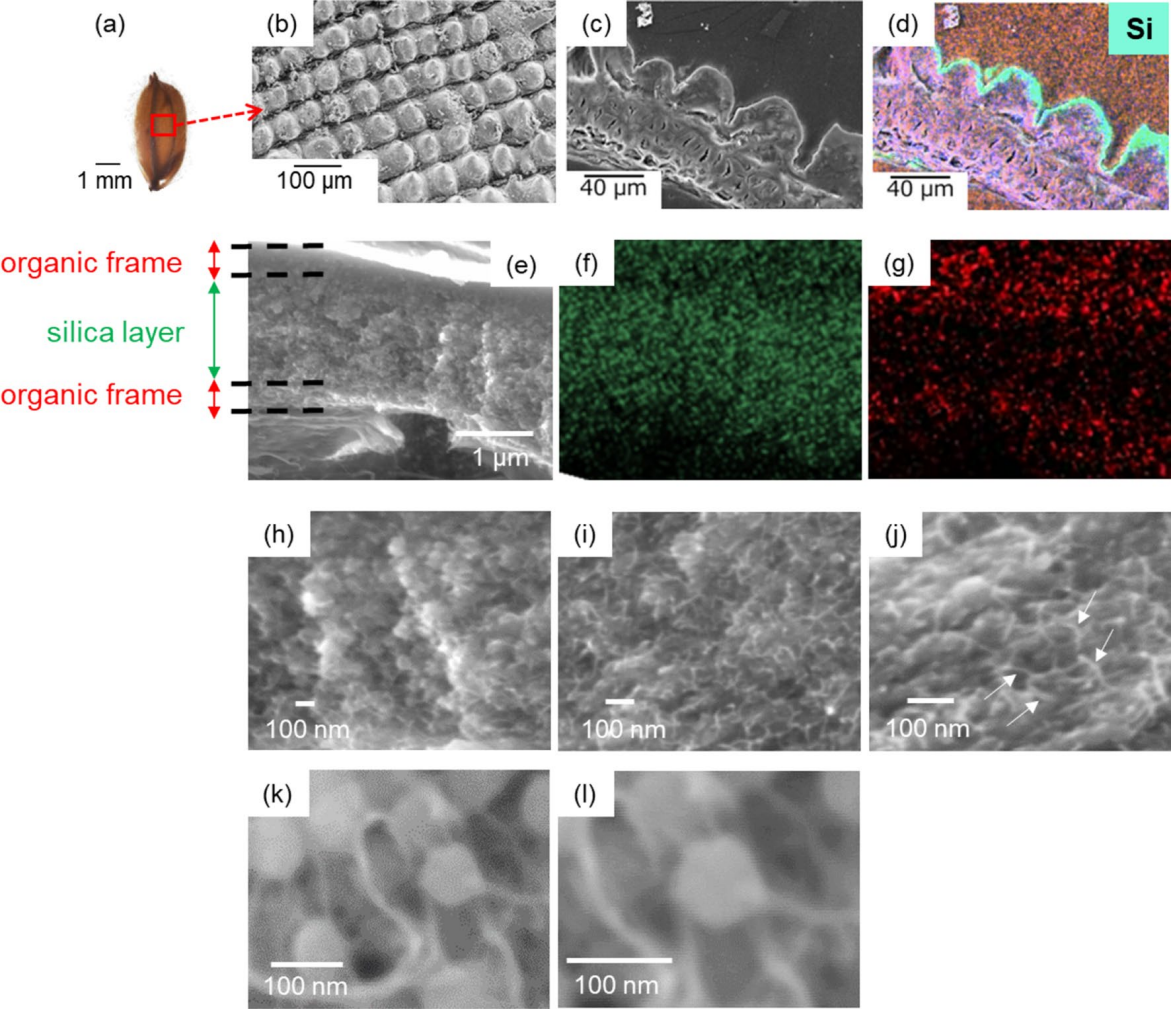
Characterization of the surface layer of a mature rice husk. A photo of a rice husk (**a**), SEM images of the surface (**b**), and the cross section (**c**, **e**, **h–l**) with elemental mapping (**d**, **f**, **g**) of a raw mature rice husk (Si: green; C: red; O: blue). White arrows indicate thin fibrils (**j**).

In individual plants, basically, husks at the tip are more mature than those at the root of a cob (Figure S2 in the SI). Figures 1 and S3 in the SI show photographic and scanning electron microscope (SEM) images of a raw mature rice husk. The convexo-concave surface of a husk was found to be covered with a silica layer 2–4 µm thick based on the presence of Si and O in the elemental mapping image (Fig. 1b–d). Enlarged cross-sectional SEM images with elemental mapping of the surface silica of a raw mature rice husk indicate that the silica layer was sandwiched between the upper and lower organic frames (Fig. 1e–g). The outside and inside organic frames are regarded as a cuticle layer and an epidermal cell wall, respectively. As shown in Fig. 1k,l, nanoparticles ~ 80 nm in diameter were observed in the silica layer of a raw husk. Intriguingly, thin fibrils ~ 10 nm wide were found to be incorporated into the silica nanoparticles in enlarged images (Fig. 1i–l).

**Figure 2:**
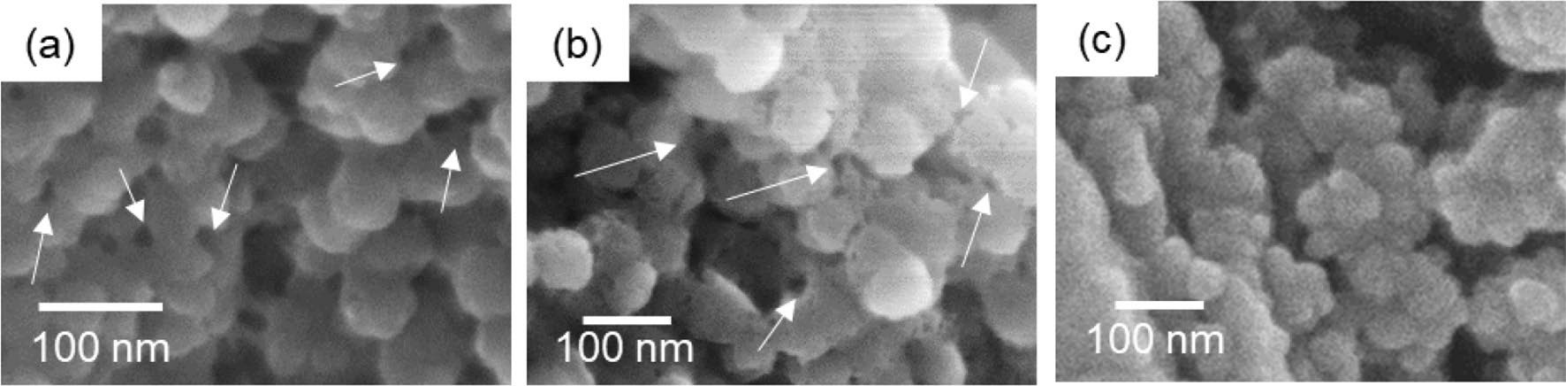
SEM images of silica particles in the surface layer of an immature rice husk after treatment with cellulase (**a**) and after treatment with the ionic liquid (**b**), and after calcination (**c**). White arrows indicate holes that were formed after dissolution of the CNFs.

The silica layer was treated with cellulase and an ionic liquid, *N*,*N*-diethyl-*N*-(2-methoxyethyl)-*N*-methylammonium 2-methoxyacetate, which dissolves cellulose specifically. As shown in Fig. 2a, b, we found silica particles 50–80 nm in diameter with removal of the fibers. Here, many holes 10–20 nm wide remained as a trace of the fibers. Thus, the thin fibrils in the silica layers are deduced to be CNFs. The remaining holes indicate that the CNFs are incorporated into the aggregation of silica nanoparticles.

Calcination also removed CNFs in the silica layers (Fig. 2c). The silica grains were smaller than those shown in Fig. 2a, b. This suggests that the silica particles observed after treatments with cellulase and the ionic liquid are enveloped by organic substance other than cellulose. Some proteins promoting silica polymerization were detected in the silica accumulation area in higher plants^28^. We confirmed the presence of proteins in the silica layer of a mature rice husk using the SDS-PAGE (sodium dodecyl sulfate–polyacrylamide gel electrophoresis) (Figure S4 in the SI). Thus, the intrafibrillar mineralization would be associated with specific proteins.

The porous bodies consisting of the silica particles after calcination were evaluated using the nitrogen adsorption technique (Figure S5 in the SI). The diameter of the primary grains was calculated to be ∼10 nm based on the specific surface area, 273 m^2^/g. The total pore volume after removal of the organic substance was roughly estimated to be 40%.

**Figure 3:**
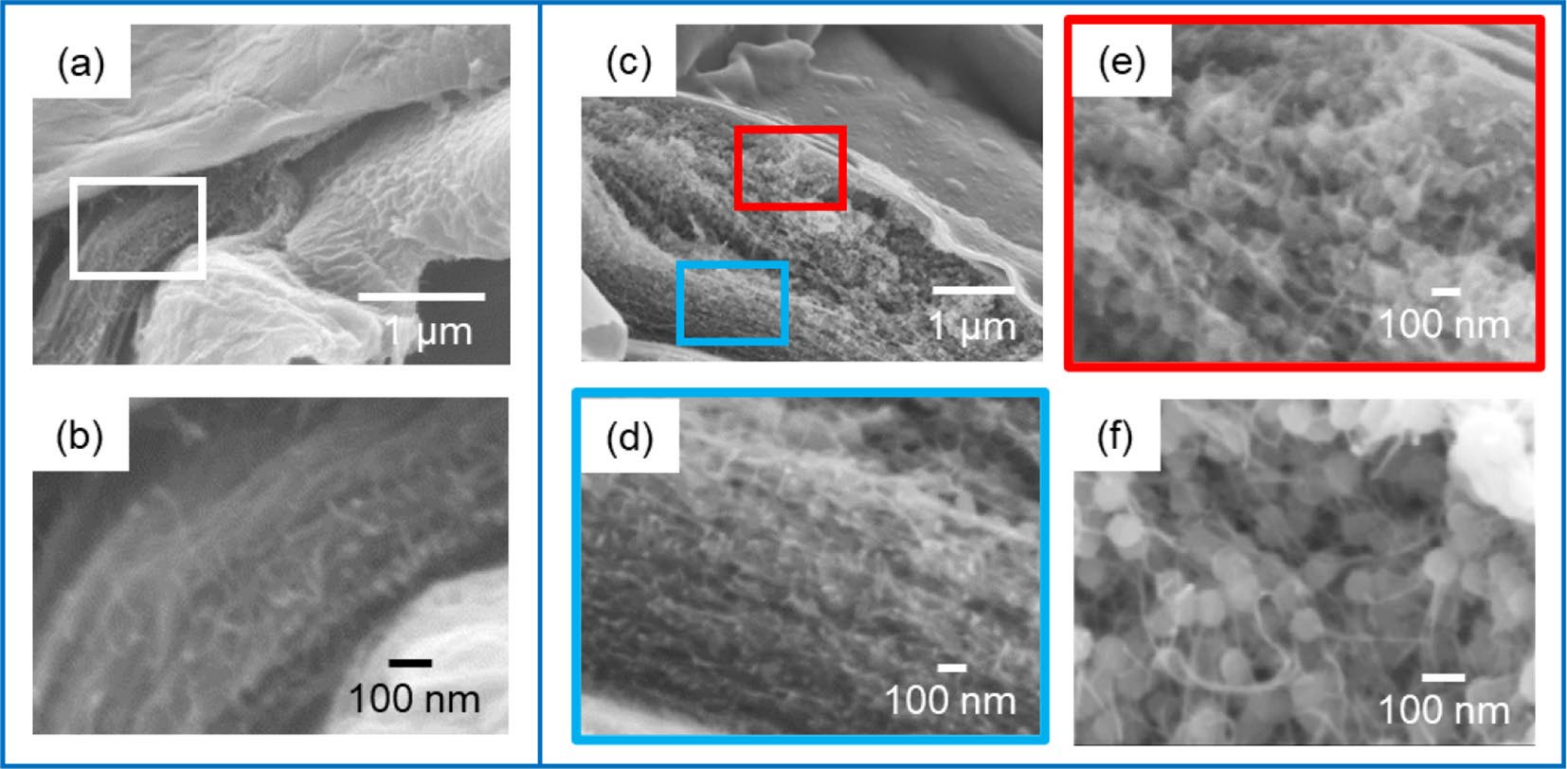
Cross-sectional SEM images of the surface layer of immature husks before (**a**, **b**) and after (**c–f**) a slight silica accumulation. The silica content was roughly estimated to be 1.3 at% (**a**, **b**) and 7.6 at% (**c–f**) using the energy dispersive X-ray spectroscopy (EDS) technique. Panels d and e show enlarged images of the blue and red frames of c, respectively.

We investigated the surface layer of immature husks to clarify the role of CNFs in the silica accumulation. Here the silicification of the silica layers was monitored by observing successional husks from the top to the root of an ear of a rice plant (Figure S2 in the SI). The husks (15–22 weeks after germination) were freeze-dried and then cut with a scalpel to observe the cross sections of the silica layer. The silica accumulation was not sufficiently recognized in the surface layer of a rice husk at the top of an ear. Figure 3a, b shows a laminated fibrous matrix in a gap between the cuticle layer (outside organic frame) and the epidermal cell wall (inside organic frame). As the silica accumulation proceeded, silica particles were formed in the fibrous matrix with the increasing distance between the two organic frames (Fig. 3c–e). The particles closer to the outside organic frame were larger than those near the inside organic frame. The organic fibers clung around the silica particles (Fig. 3f).

### Surface silica plates of rice leaf blades

**Figure 4:**
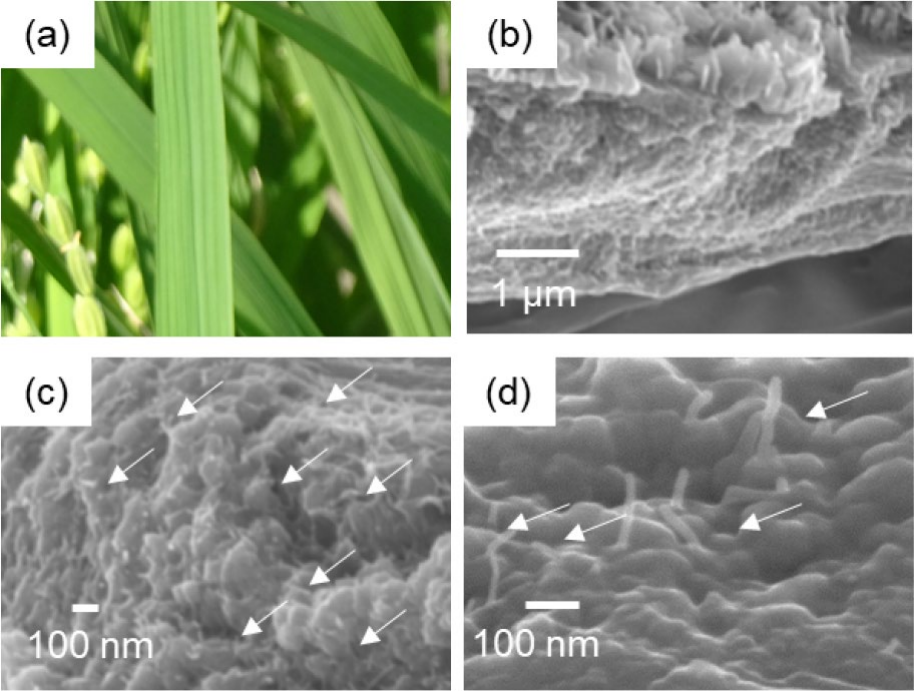
A photo of rice blades (**a**) and cross-sectional SEM images (**b–d**) of a mature rice leaf blade. The silica plate shown in panels b and c was freeze-dried and then fractured with a scalpel. White arrows indicate thin fibrils (**c**, **d**).

We investigated the presence of a fibrous matrix in the surface silica plates of a mature leaf blade (Fig. 4). In individual plants, leaf blades in the outer and tip parts are more mature than those in the inner and root parts (Figure S2 in the SI). The cross section of the tip of a freeze-dried leaf blade was exposed by fracturing with a scalpel (Fig. 4b–d). As shown in Fig. 4c, d, silica nanoparticles ~ 100 nm in diameter with thin fibrils 10–20 nm wide were observed in a raw leaf blade.

**Figure 5:**
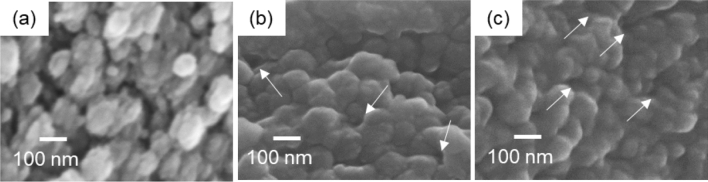
SEM images of silica particles in the surface plate of a mature leaf blade after calcination (**a**) and treatments with cellulase (**b**) and the ionic liquid (**c**). White arrows indicate holes that were formed after dissolution of the CNFs.

As shown in Fig. 5a, the fibrous matrix was removed by the calcination. We then observed that the silica particles ~ 100 nm in diameter were composed of primary grains 20–40 nm in diameter. The fibers were also removed after the cellulase and ionic liquid treatments without the deformation of silica particles, and we observed many holes in the aggregation of silica particles after removal of the CNFs (Fig. 5b, c). In a fashion similar to that in the surface layers of husks, the silica particles in the silica plates of leaf blades are comprised of small primary grains that are incorporated with the CNFs.

**Figure 6:**
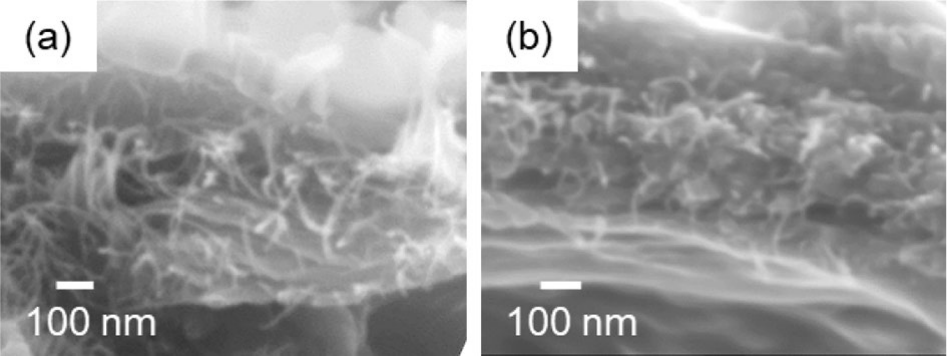
Cross-sectional SEM images of the surface layer of leaf blades before (**a**) and after (**b**) silica accumulation. We observed the surface layers at the root (**a**) and the tip (**b**) of a leaf blade that was cultured for about 1 month on a cotton bed.

Figure 6 shows cross-sectional SEM images of a leaf blade before and after the silica formation. This leaf blade was cultured for about 1 month on a cotton bed without specific silica sources. We found the fibrous matrix between the cuticle layer and the epidermal cell wall (Fig. 6a). As the leaf blade grew (Fig. 6b), silica particles with a diameter of ~ 100 nm formed between the nanofibers. The entangled structure of fibers and particles suggests that the silica plates are formed with a scaffold consisting of CNFs in the surface layer of leaf blades.

### A schematic model for cellulose intrafibrillar mineralization of plant opals

**Figure 7:**
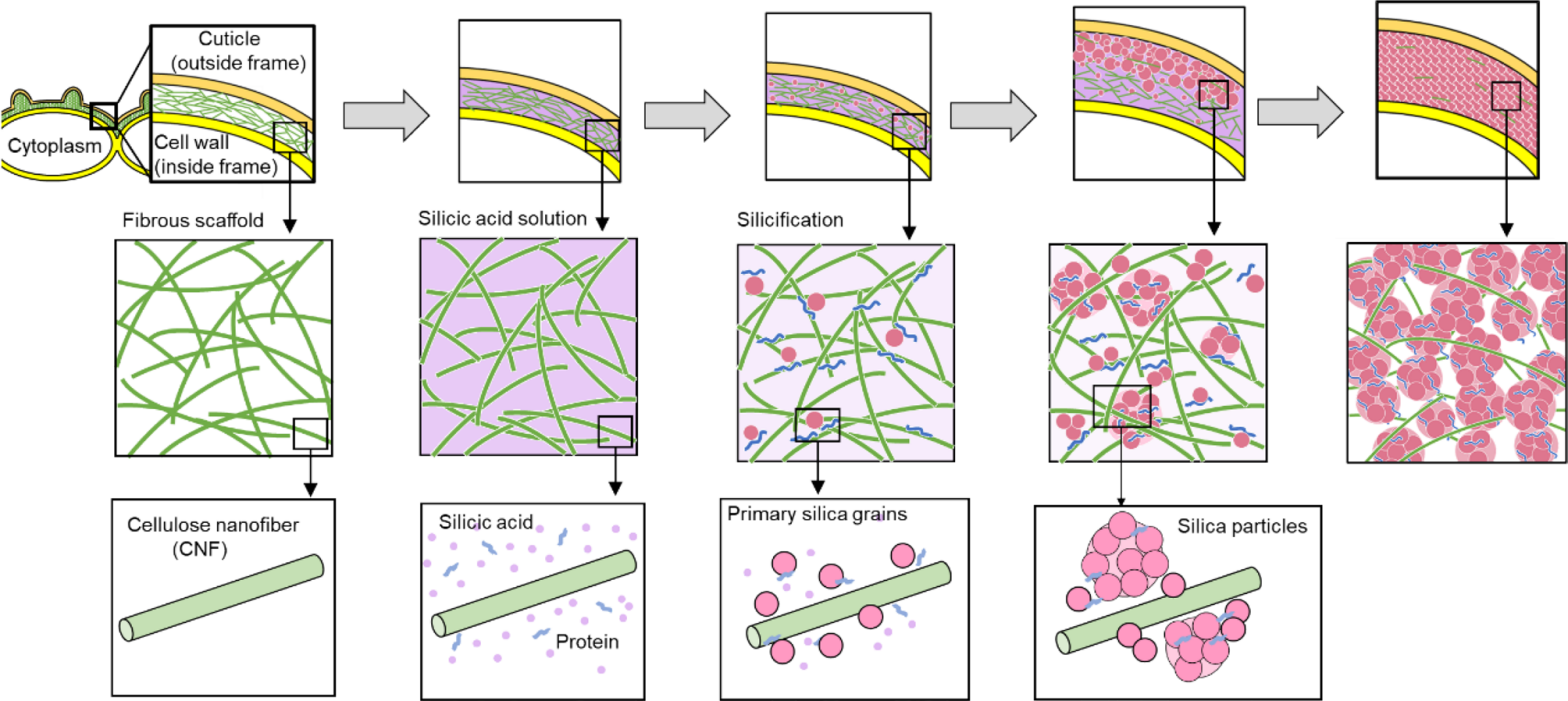
A schematic illustration of the silica accumulation process with a scaffold of CNFs. A scaffold of CNFs is initially formed as a spatial template for silicification between the cuticle layer and the epidermal cell wall. Silicic acid in the CNF matrix is polymerized with the molecular template and forms silica particles. The silica particles filled up the space between the cuticle layer and the epidermal cell wall with the CNF scaffold.

Based on our observation of the surface layer of rice husks and leaf blades, we propose the intrafibrillar mineralization process of biosilicas in a rice plant as shown in Fig. 7. A scaffold of CNFs is initially formed as a spatial template for silicification between the cuticle layer and the epidermal cell wall. The presence of CNFs was reported to influence the growth behavior of silica particles in a silicic acid solution^38^. Moreover, silica polymerization-promoting factors, such as specific proteins and long-chain polyamines synthesized in the cell, are secreted as molecular templates in the apoplast, as described in a previous report on silicification in sorghum^28^. Actually, we found several proteins in the silica layer of rice husks (Figure S4 in the SI). Thus, silicic acid in the CNF matrix is polymerized and forms silica particles with the proteins as a molecular template. The silica particles filled up the space between the cuticle layer and the epidermal cell wall with the CNF scaffold. A densely packed silica layer consisting of nanoparticles is finally formed by the silica accumulation with the increasing interframe distance.

## Conclusion

We focused on the essence of cellulose nanofibers (CNFs) with regard to silica accumulation in a rice plant. The mature plant opals in the surface layers of husks and leaf blades were found to be composed of silica particles that are incorporated with CNFs. The fibrous scaffolds are initially formed in the apoplast between the epidermal cell wall and the cuticular layer. Silica nanoparticles then fill in the gaps of the CNFs. This suggests that CNFs are involved as a scaffold in the accumulation of silica particles. The shape-controlled silicification using CNF scaffolds promotes the production of various silica bodies with specific macroscopic shapes. The role of the fibrous matrix is similar to that of collagen fibers in vertebrate bones and teeth with hydroxyapatite nanocrystals and that of chitin fibers in the crayfish gastrolith with amorphous calcium carbonate. Thus, the intrafibrillar mineralization is widely utilized for the formation of shape-controlled bodies in biomineralization.

## Experimental

Leaf blades, husks, and cobs of rice plants (*Oryza sativa* L.) were periodically sampled from 1 to 22 weeks after germination. The samples were collected from a paddy field owned by one of the authors (H. I.). Obtained samples were freeze-dried to maintain the cell structures. Since we paid attention to silicification, the degree of maturity was monitored based on the silicon content and evaluated using energy dispersive X-ray spectroscopy (EDS, JEOL JSM-7100F). In individual plants, basically, leaf blades in the outer and tip parts are more mature than those in the inner and root parts. Husks at the tip are more mature than those at the root of a cob (Figure S2 in the SI). We removed organic substances by two-step calcination as follows: dried samples were initially calcinated in an electric furnace at 350 °C in air for 90 min and then washed with purified water to remove alkaline components that deform the macroscopic morphology with subsequent calcination at a higher temperature. Finally, we calcinated the samples at 400 °C for 8 h to burn out residual organic substances. The untreated and calcinated samples were cut with a scalpel to expose their cross sections. The sample surfaces were coated with OsO_4_ and then observed using field emission scanning electron microscopy (SEM, JEOL JSM-7100F, Hitachi SU8010) at an accelerating voltage of 5 kV. The specific surface area and pore volume of the calcined samples were estimated based on the nitrogen adsorption isotherms (Micromeritics 3Flex-3MP) using the Brunauer–Emmett–Teller (BET) method. In order to identify the organic fiber, untreated samples were reacted with a specific ionic liquid, *N*,*N*-diethyl-*N*-(2-methoxyethyl)-*N*-methylammonium 2-methoxyacetate (Wako Pure Chemical), that preferentially dissolves cellulose, at 100 °C for 10 min. Freeze-dried samples were added to a phosphate buffer solution (pH 5.5) to dissolve cellulase (Onozuka RS, SERVA Electrophoresis). The parts composed of cellulose were removed by soaking in the solution at 60 °C for 2 days.

## Supplementary Information

Supplementary Information
