# Supplementary material for: Cellulose intrafibrillar mineralization of biological silica in a rice plant
Source: Sci Rep. 2021 Apr 12;11:7886. doi: 10.1038/s41598-021-87144-8 (PMC8042044; doi:10.1038/s41598-021-87144-8)
Supplement: Supplementary file 1 — Supplementary Information [file 41598_2021_87144_MOESM1_ESM.docx]

Supporting Information

**Cellulose Intrafibrillar Mineralization of Biological Silica in a Rice Plant**

Eri Nakamura^1^・Noriaki Ozaki^2^・Yuya Oaki^1^・Hiroaki Imai^1*^

1: Department of Applied Chemistry, Faculty of Science and Technology, Keio University, 3-14-1 Hiyoshi, Kohoku-ku, Yokohama 223-8522, Japan /E-mail^※^: hiroaki@applc.keio.ac.jp

2: Department of Biotechnology, Faculty of Bioresource Sciences, Akita Prefectural University, 241-438 Kaidobata-Nishi, Nakano Shimoshinjo, Akita 010-0195, Japan


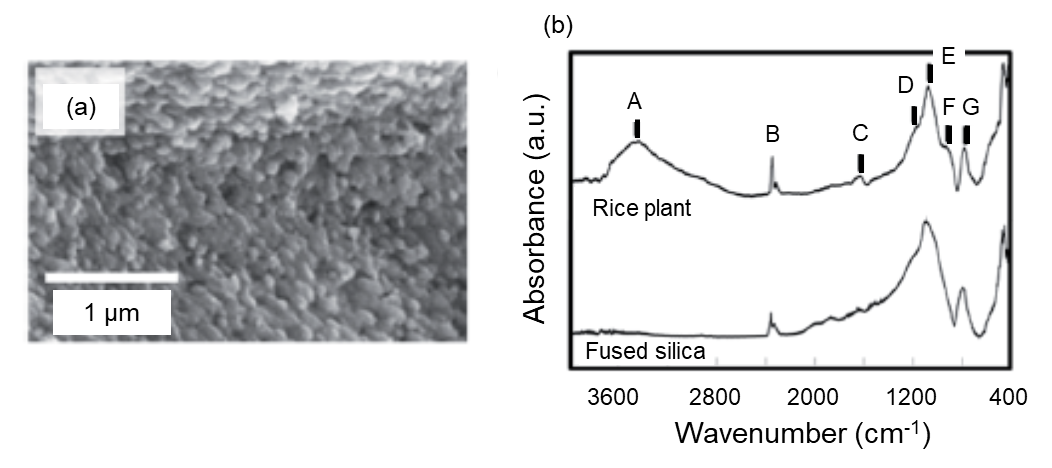


(a)


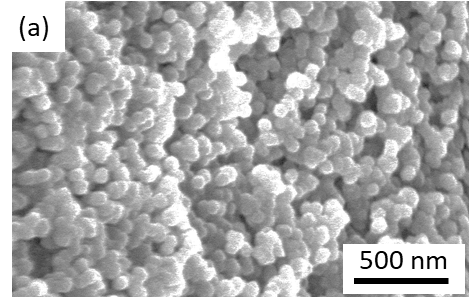


Figure S1. SEM image of rice leaf blades of the inner surface (a) and Fourier transform infrared absorption spectra (b) of the plant opals and fused silica. (A: The O–H stretching mode; B: the stretching mode of CO_2_; C: the H–O–H bending mode; D: the Si–O–Si stretching TO mode; E: the Si–O–Si stretching LO mode; F: the Si–OH stretching mode; G: the Si–O–Si bending TO mode). Reproduced from Ref. #1 with the permission of the Royal Society of Chemistry. The presence of a specific absorption band (D, E, G) indicates that the nanoparticles are composed of silica. The broad and intense signal A originating from the O–H stretching mode for the plant opals is ascribed to the presence of a large amount of OH groups on the grain surface.


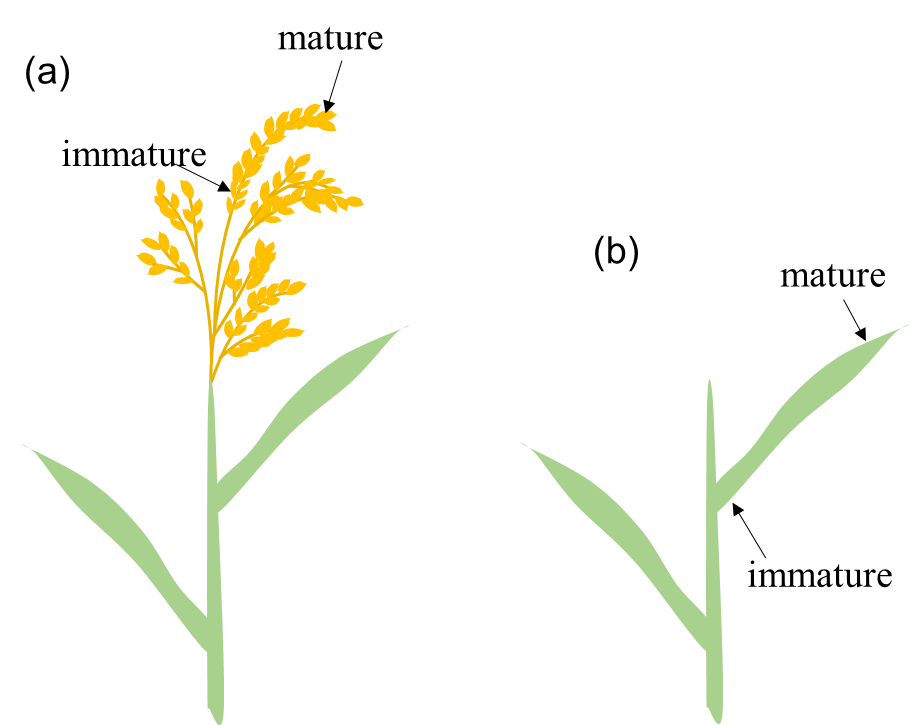


Figure S2. Schematic illustrations of matured and unmatured parts of rice husks (a) and leaf blades (b).


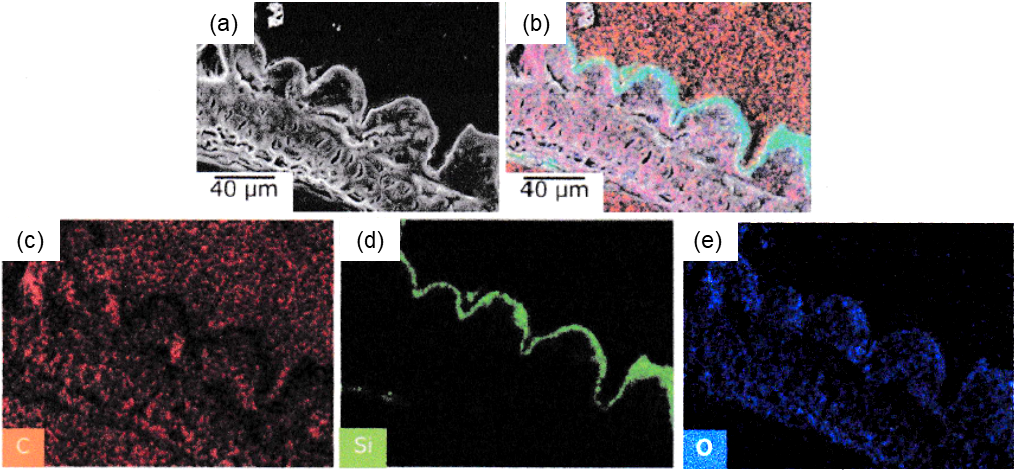


Figure S3. A cross-sectional SEM image (a) with elemental mapping (C (c), Si (d), O (e), and superposition (b)) of the surface silica of a raw mature rice husk.

**
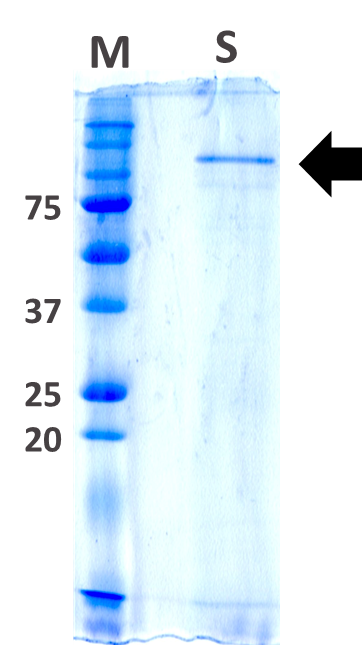
**

Figure S4. Electrophoresis of HF-soluble fractions of mature rice husks. M: Molecular weight marker (Precision Plus Protein Dual Xtra Standards, BIORAD); S: soluble proteins by HF extraction; arrow indicates a major protein band stained with Coomassie Brilliant Blue (CBB).

Rice husks were freeze-dried prior to the extraction of proteins. For cell-wall protein extraction, the rice husks were soaked in an SDS-PAGE sample buffer containing 1% SDS / 50 mM dithiothreitol (DTT) and heated for 5 min at 95 ℃. After washing several times, the resulting residue was suspended in 4 M hydrogen fluoride (HF) solution to dissolve silicas and left for 2 h at room temperature. After centrifugation for 10 min at 2000 g, the supernatant was submitted to dialysis with distilled water and lyophilized. The sample was subjected to SDS-PAGE, and gels were stained with Coomassie Brilliant Blue (CBB; EzStain AQua, ATTO). Only one major band (arrow) was visualized with CBB. The amino acid sequence of this protein is currently under preparation for submission, but part of the sequence is relatively rich in pairs where basic and acidic amino acids are adjacent, for example, in arginine-aspartic acid.


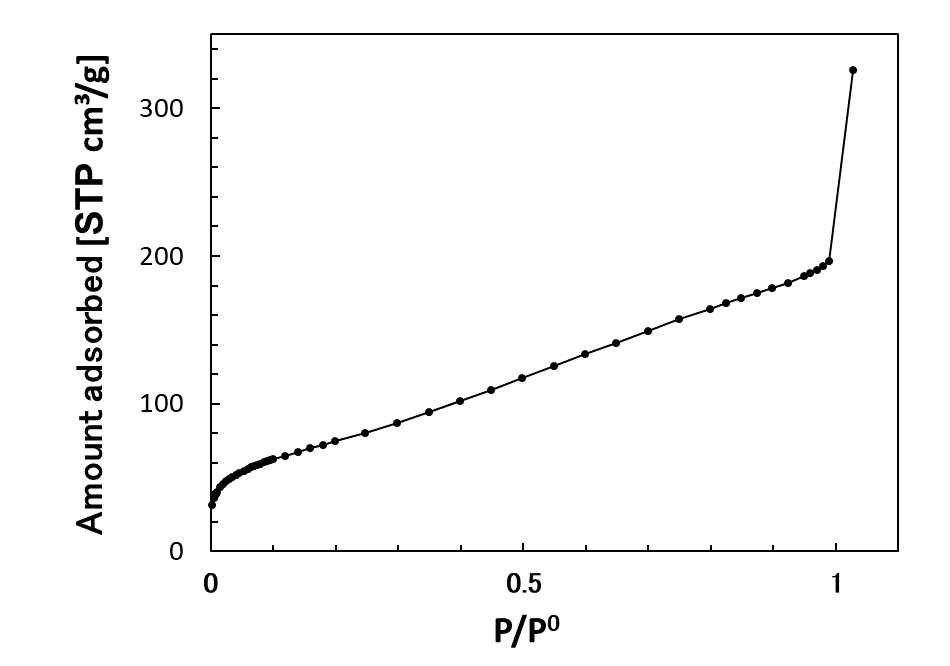


Figure S5. Nitrogen adsorption isotherm for the silica layer of a rice husk after calcination.

**Reference**

1. Hoshino, T.; Sato, K.; Oaki, Y.; Sugawara-Narutaki, A.; Shimizu, K.; Ozaki, N.; Imai, H. Plant opal-mimetic bunching silica nanoparticles mediated by long-chain polyethyleneimine. *RSC Adv.* 6, 1301–1306 (2016).
